# A Natural Mouse Model for Neisseria Colonization

**DOI:** 10.1128/IAI.00839-17

**Published:** 2018-04-23

**Authors:** Mancheong Ma, Daniel A. Powell, Nathan J. Weyand, Katherine A. Rhodes, María A. Rendón, Jeffrey A. Frelinger, Magdalene So

**Affiliations:** a Department of Immunobiology and BIO5 Institute, University of Arizona, Tucson, Arizona, USA; b Valley Fever Center for Excellence, University of Arizona, Tucson, Arizona, USA; University of Texas at Austin

**Keywords:** commensalism, commensal and pathogenic Neisseria, type IV pilus, Collaborative Cross, host restriction of colonization, innate immunity, commensal and pathogenic Neisseria

## Abstract

Commensals are important for the proper functioning of multicellular organisms. How a commensal establishes persistent colonization of its host is little understood. Studies of this aspect of microbe-host interactions are impeded by the absence of an animal model. We have developed a natural small animal model for identifying host and commensal determinants of colonization and of the elusive process of persistence. Our system couples a commensal bacterium of wild mice, Neisseria musculi, with the laboratory mouse. The pairing of a mouse commensal with its natural host circumvents issues of host restriction. Studies are performed in the absence of antibiotics, hormones, invasive procedures, or genetic manipulation of the host. A single dose of N. musculi, administered orally, leads to long-term colonization of the oral cavity and gut. All mice are healthy. Susceptibility to colonization is determined by host genetics and innate immunity. For N. musculi, colonization requires the type IV pilus. Reagents and powerful tools are readily available for manipulating the laboratory mouse, allowing easy dissection of host determinants controlling colonization resistance. N. musculi is genetically related to human-dwelling commensal and pathogenic Neisseria and encodes host interaction factors and vaccine antigens of pathogenic Neisseria. Our system provides a natural approach for studying Neisseria-host interactions and is potentially useful for vaccine efficacy studies.

## INTRODUCTION

Commensals (i.e., microbiota) play a critical role in the physiology of multicellular organisms. They are required for the homeostasis of many bodily processes, and they participate in gut and immune system development and prevent pathogen colonization. Perturbations in these microbial communities are strongly linked to obesity, inflammatory bowel disease, diabetes, and autoimmunity (1–7).

The mechanisms underlying host and commensal determinants of persistent colonization are little understood. The majority of commensals cannot be cultured or manipulated genetically (8–10). Because of host restriction barriers, few animal models provide a natural setting for probing commensal-host interactions. Neisseria, a genus of Gram-negative betaproteobacteria, provides an opportunity to develop a natural small animal model for this purpose.

The genus Neisseria contains a large number of genetically related species (11). The vast majority of these are commensals of hosts ranging from rodents, canids, and bovines to nonhuman primates and humans (12–17). Neisseria gonorrhoeae and Neisseria meningitidis are the only two species that cause disease. These pathogens, which infect only humans, also behave like commensals in that they have a tendency to colonize asymptomatically (18–20). Commensal Neisseria spp. are little studied, and there are no small animal models for colonization. Several mouse models have been developed for pathogenic Neisseria infection, but due to the strict tropism of N. gonorrhoeae and N. meningitidis for humans, they are necessarily heterologous systems that require invasive procedures; antibiotics; hormones; direct administration of human homologous proteins, such as transferrin; and/or the use of transgenes expressing human proteins (21–24).

We recently isolated a new species of commensal Neisseria, Neisseria musculi, from the oral cavity (OC) of healthy wild mice (17). N. musculi is easily cultured and manipulated *in vitro* and is genetically related to other Neisseria. With the aim of developing a small animal model for Neisseria colonization, we determined whether N. musculi could be paired with inbred laboratory mice. We report that N. musculi colonizes the oral cavity and gut of laboratory mice for at least 1 year without causing disease. Long-term colonization is achieved with a single oral dose. Using this model, we discovered that permissiveness to N. musculi colonization is strongly influenced by host genetics and by innate, but not adaptive, immunity. For N. musculi, colonization requires its type IV pilus (Tfp). Finally, we present evidence that N. musculi encodes homologs of host interaction factors and vaccine antigens found in pathogenic Neisseria spp. and that it expresses one of the vaccine targets, capsular polysaccharide. We discuss the power of our natural small animal model to broaden our knowledge of commensal and pathogenic Neisseria biology and of host components that restrict/permit colonization.

## RESULTS

### N. musculi colonizes the oral cavities and guts of mice in a mouse strain-specific manner.

We isolated N. musculi from the oral cavity of a wild mouse, Mus musculus domesticus (17). Our repeated attempts to culture Neisseria from the oral cavities of inbred mice from Jackson Laboratory and Taconic were unsuccessful. Since inbred laboratory mice do not harbor Neisseria, this provided an opportunity to test the susceptibility of these animals to N. musculi colonization.

The Collaborative Cross (CC) is a powerful new tool in mouse genetics that allows the linkage of alleles with phenotypic traits (25). We tested N. musculi on selected CC founder strains. These strains include 5 conventional, widely used inbred strains and 3 wild-derived inbred strains from distinct M. musculus subspecies (Table 1). The wild-derived strains are CAST, wild mice trapped in Thailand belonging to a distinct subspecies, Mus musculus castaneous; PWK, trapped in the Czech Republic and belonging to the subspecies Mus musculus musculus; and WSB/EiJ (WSB), trapped in Maryland, USA, and belonging to M. musculus domesticus. The conventional inbred strains are chimeras with varying degrees of genetic relatedness to CAST, PWK, and WSB, although their genetic origins are overwhelmingly M. musculus domesticus (25).

**TABLE 1:** Susceptibility of Collaborative Cross founder strains to colonization by N. musculi

Strain	No. colonized/inoculated (%)^*a*^	*P* value^*b*^
CAST/EiJ	35/40 (87)	
A/J	26/28 (92)	NS^*c*^
C57BL/6J	12/23 (52)	<0.006^*c*^
NOD/LtJ	0/4 (0)	<0.0004^*c*^
NZO/HILtJ	0/4 (0)	<0.0004^*c*^
PWK/PhJ	0/9 (0)	<10^5^^*c*^
WSB/EiJ	0/9 (0)	<10^5^^*c*^
129S1/SvImJ	0/4 (0)	<0.0004^*c*^
MyD88^−/−^	21/21 (100)	<0.001^4^
RAG-1^−/−^	3/14 (21)	NS^*d*^

a Mice were scored for the presence of N. musculi in the oral cavity and fecal pellet each week for 3 months.

b χ^2^ with Yates correction for small numbers and Bonferroni for multiple pairwise comparisons. NS, not significant.

c Compared to CAST.

d Compared to WT BL/6.

The mouse inoculation protocol is shown in Fig. S1 in the supplemental material. Prior to inoculation, the presence of Neisseria in these animals was determined by plating OC and fecal pellet (FP) samples on selective agar. The mice used in this study have always been culture negative. The next day, AP2365, a naturally occurring rifampin-resistant (Rif^r^) rough variant of N. musculi, was gently pipetted into the OCs of the animals, and N. musculi counts in OCs and FPs were determined weekly for 3 months by plating samples on selective agar. CAST/EiJ (CAST) and A/J mice were very susceptible to colonization (Table 1): the OCs and FPs of 35/40 (87%) CAST mice and 26/28 (92%) A/J mice were continuously culture positive. C57BL/6J (B6) mice (12/23; 52%) were partially resistant to colonization. In contrast, NOD, NZO, PWK, WSB, and 129S1 mice were highly resistant at the same infectious dose by the same route of inoculation.

**FIG 1:**
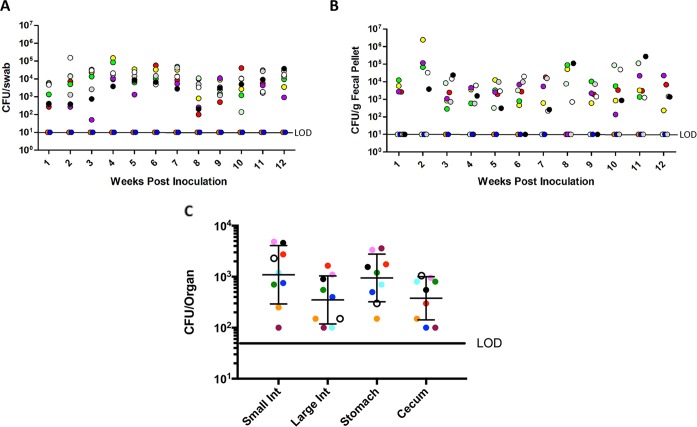
N. musculi colonizes the oral cavity (A) and gut (B) of CAST mice and different sections of the gastrointestinal tract (C). The samples in panels A and B are from the same experiment; each mouse was assigned a unique color. The samples in panel C are taken from 3-month-colonized CAST mice from a different experiment. The plots indicate geometric means with geometric standard deviations (SD). LOD, limit of detection; Int, intestine.

A representative colonization experiment, involving inoculation of 10 CAST mice, is shown in Fig. 1. The number of N. musculi CFU in the OC and FP quickly reached a plateau and remained steady thereafter, indicating the commensal had adapted to these niches and that replication and turnover had reached equilibrium. Generally, when N. musculi was cultured from the OC, it was also recovered from the FP. Samples reisolated from colonized mice were N. musculi, as judged by multilocus sequence typing of 51 ribosomal genes (rMLST) of 10 OC and 10 FP colonies recovered from CAST mice at 5 weeks postinoculation (data not shown). All N. musculi bacteria reisolated from the OC and FP had the rough colony phenotype, like the inoculation strain.

Two colonized CAST mice were followed long term. N. musculi was continuously recovered from their OCs and FPs for 52 weeks (see Fig. S2 in the supplemental material). Colonized B6 mice yielded similarly high N. musculi counts weekly for 52 weeks (data not shown).

Throughout our studies, all inoculated and uninoculated mice remained healthy: none lost weight, and all maintained healthy coats and normal activity. At necropsy (performed by D. Beselson, Director, University of Arizona Animal Care Facility), the organs of the 52-week-colonized mice resembled those of healthy mice.

N. musculi was not cultured from the peripheral blood of 4 CAST and 4 A/J mice 4 h or 28 days postinoculation. Although this experiment did not address whether N. musculi enters the bloodstream, the result suggests the commensal did not survive at this site.

Taken together, these results demonstrate that the susceptibility of a mouse to N. musculi colonization is strongly influenced by its genetic background. In susceptible mouse strains, N. musculi easily colonizes the OC and gut and persists in these niches for lengthy periods without causing disease.

### N. musculi colonizes the entire gastrointestinal tract in mice.

We examined the location of N. musculi in the gastrointestinal tracts of 3-month-colonized CAST mice. The stomachs, small intestines, large intestines, and ceca of necropsied animals were flushed with sterile saline to remove the luminal content, and the tissues were homogenized and plated on selective agar. N. musculi was recovered from all sampled sections of the gut (Fig. 1C). It was impossible to sample organs from the same animal on successive days or to determine whether N. musculi populations in these organs were self-sustaining. However, the large numbers of N. musculi bacteria recovered from tissue-associated gut samples long after inoculation strongly suggest the commensal was not simply in transit from the OC.

To determine whether N. musculi could be horizontally transmitted, we cohoused 2 colonized CAST mice with 3 naive CAST or B6 mice for 12 weeks. None of the uninoculated mice became colonized. To determine whether the endogenous flora influenced colonization, we cohoused 4 B6 and 4 CAST mice for 12 weeks before inoculation. This did not alter the colonization susceptibility of either mouse. Moreover, CAST and B6 mice bred in house or purchased from the Jackson Laboratory were always colonized at the same frequency. Although these experiments involved small numbers of mice, the evidence suggests that the preexisting flora did not play a significant role in determining colonization susceptibility; final evidence awaits fecal transplant studies. To determine whether *in vivo* passage of N. musculi would increase its colonization efficiency, we inoculated 4 naive B6 mice with N. musculi isolated from the OC of a persistently colonized CAST mouse. This *in vivo* passage did not alter N. musculi colonization efficiency. Taken together, these results suggest that neither housing conditions nor the endogenous microbiota is a significant roadblock to N. musculi colonization.

### Innate immunity determines susceptibility to N. musculi colonization.

**FIG 2:**
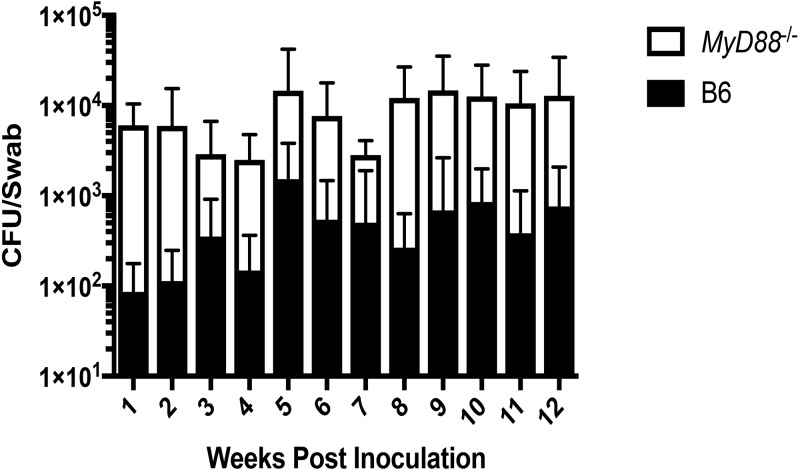
MyD88^−/−^ mice have higher N. musculi burdens than parental B6 mice. N. musculi CFU in oral swabs taken from B6 and MyD88^−/−^ mice are shown (*n* = 9 or 10 mice/group). The bars indicate means with SD. Significance was determined using Student's *t* test on the average burden per strain over the lifetime of the experiment. The data are representative of 2 independent experiments.

The partial resistance of B6 mice to N. musculi colonization (Table 1) provided an opportunity to investigate the role of the immune system in determining colonization susceptibility. N. musculi was assayed in two strains of immunodeficient B6 mice: B6-MyD88^−/−^ mice, which lack the MyD88 adaptor that mediates signaling through many Toll-like receptors, and B6-Rag-1^−/−^ mice, which lack T and B cells and cannot mount an adaptive immune response (Table 1). Strikingly, MyD88^−/−^ mice were exquisitely susceptible to N. musculi colonization, unlike the B6 parental strain (21/21 MyD88^−/−^ mice colonized versus 12/23 B6 mice; *P* < 0.001). MyD88^−/−^ mice also had higher N. musculi burdens than the parental wild-type (WT) strain (*P* = 0.0026) (Fig. 2). In contrast, Rag-1^−/−^ mice were no more susceptible than WT B6 mice. These results indicate that the innate, but not the adaptive, immune system is a major determinant of N. musculi colonization. The increased numbers of N. musculi bacteria recovered from MyD88^−/−^ mice compared to WT B6 mice suggest that the innate response plays an ongoing role in controlling N. musculi numbers.

### N. musculi colonization requires the type IV pilus.

To test the usefulness of our model for studying commensal determinants of colonization, we focused our attention on the Tfp. All Neisseria species have a complete set of Tfp biogenesis genes (16, 17, 26). In the case of pathogenic Neisseria, Tfp is implicated in promoting colonization, based on experiments using cultured human cells and a limited number of human challenge studies (27–31). The function of Tfp has never been tested in a natural animal model.

**FIG 3:**
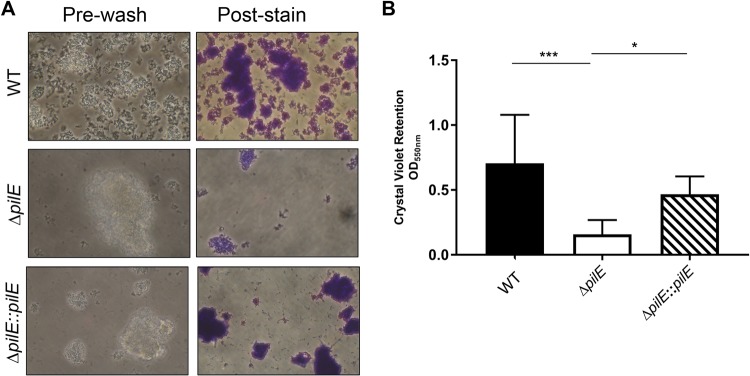
N. musculi Δ*pilE* is defective in attachment (A) and biofilm formation (B). Δ*pilE*::Δ*pilE*, complemented strain. (B) Statistical analysis was performed in GraphPad Prism 7 by one-way analysis of variance (ANOVA) with Tukey's multiple-comparison test. ***, *P* < 0.001; *, *P* < 0.05. No significant difference was detected between the WT and complemented strains.

For this experiment, a nonpiliated mutant of N. musculi, Δ*pilE*, was constructed by deleting the gene encoding the Tfp fiber subunit; a complemented strain, Δ*pilE*::*pilE*_WT_-C10, was also constructed. The piliation statuses of the Δ*pilE* and complemented strains were validated by several methods. Unlike the WT and complemented strains, Δ*pilE* did not produce *pilE* mRNA, as judged by reverse transcription (RT)-PCR (see Fig. S3 in the supplemental material). N. musculi Δ*pilE* exhibited phenotypes characteristic of nonpiliated mutants: it was defective in DNA transformation (see Table S1 in the supplemental material) and attached less well to surfaces (Fig. 3). These results indicate N. musculi Δ*pilE* does not produce the Tfp fiber. Finally, the growth of Δ*pilE* was examined. The WT, Δ*pilE*, and complemented strains grew equally well (see Fig. S4 in the supplemental material). The slightly lower optical density at 600 nm (OD_600_) of Δ*pilE* cultures was not statistically different at any time point; it likely reflects the slight tendency of Δ*pilE* cells to aggregate in liquid culture.

**FIG 4:**
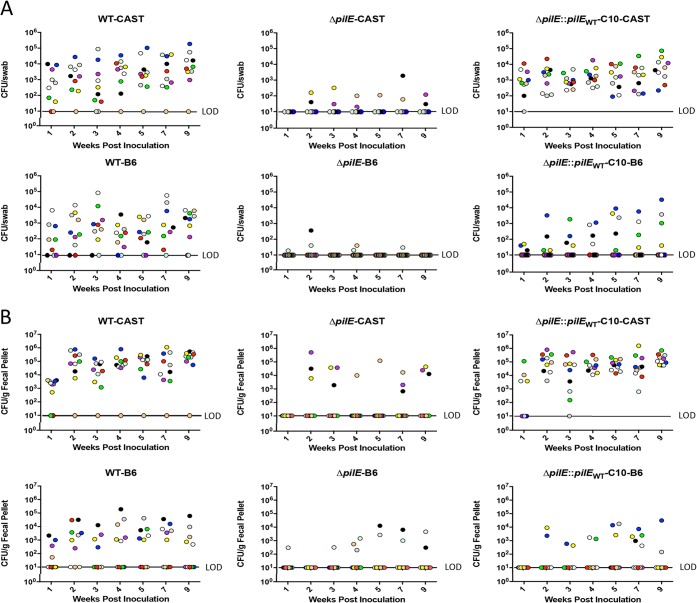
N. musculi Δ*pilE* is defective in colonizing the oral cavity (A) and gut (B) of CAST and B6 mice. AP2365 Δ*pilE*::*pilE*_WT_-C10, *pilE* complemented strain. Each N. musculi strain was assayed in 10 mice. Oral-swab and fecal samples from the same mouse were assigned the same color.

N. musculi Δ*pilE* was defective in colonizing the OC and gut in CAST and B6 mice compared to the WT and complemented strains (Fig. 4; see Table S3 in the supplemental material) (*P* < 0.0001 for WT versus Δ*pilE* for both CAST and B6 mice). The few OC and FP reisolates were N. musculi, as judged by *pilE* sequencing (primers NP246F and NP246R2), and their mutated *pilE* locus was unaltered (data not shown). The complemented Δ*pilE*::*pilE*_WT_-C10 strain colonized the OC and FP of CAST mice like WT N. musculi (OC, *P* = 0.3316; FP, *P* = 0.9916). In B6 mice, the colonization behavior of the complemented strain did not fully revert to that of WT N. musculi, even after taking into account the partial colonization resistance of B6 mice (OC, *P* = 0.0013; FP, *P* = 0.0008). We cannot explain this behavior. During the transformation/recombination process that inserted the WT *pilE* sequence into the Δ*pilE* strain, a mutation may have occurred elsewhere in the genome that affected the colonization behavior of the complemented strain. The transformation/recombination process may have had a polar effect on a gene immediately downstream of the complemented *pilE* site. In the annotated N. musculi genome, *pilE* is at the end of the contig; the identity of the downstream gene is unknown. Other explanations are also possible, but we note that this colonization behavior of the complemented strain is observed only in the B6 genetic background and not in the CAST background. Finally, we note that in these experiments, the rough variant of N. musculi (WT, Δ*pilE*, and complemented strains) was used, and all reisolated N. musculi strains exhibited the rough colony phenotype.

### N. musculi encodes host interaction factors and vaccine candidates of human-dwelling Neisseria.

Finally, we determined whether N. musculi could be used to model human-dwelling species of Neisseria. To date, Neisseria colonization studies have focused almost exclusively on the two pathogens N. gonorrhoeae and N. meningitidis. Great efforts have been made to identify host interaction factors with the goal of identifying vaccine antigens capable of stimulating protective immune responses. Chief among these vaccine development efforts has been the use of reverse vaccinology to identify genome-derived Neisseria antigens (GNAs) in N. meningitidis (32). Currently, similar work is being conducted to identify vaccine antigens in N. gonorrhoeae (33, 34).

Many homologs of pathogenic Neisseria host interaction factors and candidate vaccine antigens, including GNAs, were found in N. musculi and human-dwelling commensal Neisseria strains (Tables 2 and 3). Two GNAs with high identity and query coverage values were GNA1220, a membrane protein of unknown function containing a stomatin-like domain; and GNA33, a membrane-associated lytic transglycosylase required for cell separation (35, 36). N. meningitidis GNA1946 and the N. gonorrhoeae ortholog, NGO2139, which are methionine-binding subunits of ABC transporters, both retrieved the same N. musculi ortholog with greater than 75% identity and 95% query coverage. GNA1946 and NGO2139 (MetQ) induce the production of serum bactericidal antibodies (32, 37). N. musculi also has a homolog for N. meningitidis LpdA, a high-molecular-weight protein, P64k, that is very immunogenic and is used frequently as a carrier protein for weaker immunogens (38, 39).

**TABLE 2:** Putative orthologs of protective antigens encoded in the AP2031 genome

Protein query	Query accession no.	Query species	Ortholog																	
			N. musculi		N. polysaccharea		N. lactamica		N. cinerea		*N. subflava*		N. oralis		N. mucosa		N. elongata		N. bacilliformis	
			% id.^*a*^	% qc.^*b*^	% id.	% qc.	% id.	% qc.	% id.	% qc.	% id.	% qc.	% id.	% qc.	% id.	% qc.	% id.	% qc.	% id.	% qc.
LctP	CBA04244	N. meningitidis	25	*95*	25	*95*	**98**	*100*	**92**	*100*	**92**	*100*	**92**	*100*	**94**	*100*	**82**	*99*	40	42
LpdA	CAA57206	N. meningitidis	**74**	*100*	**74**	*100*	**85**	*100*	**84**	*100*	**85**	*100*	**84**	*100*	**84**	*100*	**71**	*100*	**72**	*100*
GNA1030^*c*^	NP_274064	N. meningitidis	48	23	29	79	**95**	*88*	**90**	*100*	**74**	*100*	**86**	*100*	**91**	*86*	**65**	*88*	**62**	*100*
GNA1220	NP_274245	N. meningitidis	**81**	*99*	**81**	*99*	**93**	*100*	**93**	*100*	34	31	**82**	98	**84**	*100*	**75**	*99*	**78**	*96*
GNA1946	NP_274940	N. meningitidis	**77**	*95*	**77**	*95*	**88**	*100*	**85**	*100*	**79**	*97*	**82**	*97*	**82**	*98*	**80**	*96*	**67**	*96*
GNA2091^*c*^	NP_275079	N. meningitidis	**63**	*79*	**63**	*79*	**88**	*100*	**80**	*100*	**86**	*79*	**67**	*99*	**85**	*80*	**63**	*79*	**65**	*79*
GNA33	NP_273099	N. meningitidis	**77**	*85*	**77**	*85*	**91**	*100*	**87**	*100*	**78**	*100*	**73**	*99*	**77**	*100*	**74**	*85*	**67**	*94*
NadA^*c*^^,^^*d*^	NP_274986	N. meningitidis	43	15	43	15	27	30	**67**	28	ND^*e*^	ND^*e*^	38	15	**69**	46	48	7	35	27
PorA P1^*c*^^,^^*d*^	NP_273150	N. meningitidis	**53**	*97*	**53**	*97*	**86**	*100*	**85**	*100*	**71**	*98*	**59**	*98*	**71**	*100*	**61**	*99*	**56**	*99*
ExbB	NP_274732	N. meningitidis	**68**	*99*	**68**	*99*	**96**	*100*	**93**	*99*	**73**	*99*	**73**	*99*	**76**	*99*	**58**	*99*	**57**	*98*
GNA992	NP_274028	N. meningitidis	48	23	48	23	**91**	*94*	39	*79*	**62**	23	**64**	14	**65**	29	**63**	67	**64**	18
GNA2001	NP_274993	N. meningitidis	**68**	*65*	**68**	*65*	**65**	*100*	**60**	*100*	**60**	*97*	**56**	*100*	**56**	*97*	**79**	*98*	**76**	57
GNA1870 (fHbp)^*c*^^,^^*d*^	NP_274866	N. meningitidis	30	38	30	38	34	61	**91**	*100*	39	*83*	31	62	38	*82*	28	*89*	29	64
NspA	NP_273705	N. meningitidis	42	*86*	42	*86*	**84**	*87*	48	*86*	45	*86*	29	36	40	43	47	*86*	45	*86*
TBP2	CAA55541	N. meningitidis	25	13	25	13	**74**	*10*0	35	*98*	26	8	28	17	31	12	26	10	28	47
TbpA	AAF81744	N. meningitidis	30	54	30	54	**94**	*100*	75	*100*	26	58	28	61	30	71	31	67	32	54
GNA2132 (NHBA)^*c*^^,^^*d*^	NP_275117	N. meningitidis	ND^*e*^	ND	ND	ND	**75**	*100*	40	40	32	25	34	9	35	51	32	29	28	34
GNA1162	NP_274189	N. meningitidis	33	40	33	40	**95**	*100*	**88**	*100*	48	13	35	14	**54**	*99*	48	35	**52**	10
PilC1	YP_207232	N. gonorrhoeae	36	68	36	68	46	*100*	40	*89*	40	65	36	67	38	66	24	65	26	31
PilQ	YP_207267	N. gonorrhoeae	**56**	*100*	**56**	*100*	**81**	*100*	**77**	*100*	**61**	*100*	**57**	*99*	**58**	*100*	49	*97*	49	*97*
AniA	YP_208345	N. gonorrhoeae	**79**	*79*	**79**	*79*	**93**	*79*	**92**	*79*	**87**	*79*	**80**	*79*	**89**	*79*	**80**	*79*	**78**	*79*
OpaD	YP_208563	N. gonorrhoeae	32	69	32	69	**66**	*100*	29	*86*	32	*85*	30	19	23	16	32	*89*	29	*85*
OpcA	CAB45007	N. gonorrhoeae	28	16	28	16	40	64	41	*83*	25	*76*	20	*76*	31	15	26	*82*	23	38
LptD	YP_208748	N. gonorrhoeae	**60**	*98*	**60**	*98*	**91**	*98*	**81**	*100*	**63**	*98*	**61**	*98*	**61**	*98*	**55**	*100*	**55**	*92*
BamA	YP_208831	N. gonorrhoeae	**75**	*100*	**75**	*100*	**90**	*100*	**94**	*100*	**82**	*100*	**80**	*100*	**82**	*100*	**71**	*100*	**69**	*100*
TamA	YP_208979	N. gonorrhoeae	**68**	*90*	**68**	*90*	**95**	*100*	**83**	*98*	**77**	*89*	**74**	*88*	**75**	*88*	**63**	*89*	**62**	*89*
NGO2054	YP_209073	N. gonorrhoeae	**64**	*76*	**64**	*76*	**94**	*100*	**78**	*100*	**62**	*100*	**57**	*81*	**60**	57	**54**	*100*	**62**	74
NGO2139 (MetQ)	YP_209148	N. gonorrhoeae	**78**	*95*	**78**	*95*	**90**	*100*	**78**	*100*	**82**	*97*	**86**	*97*	**85**	*98*	**82**	*96*	**77**	*82*

a %id., percent identity. Boldface indicates >50% identity.

b %qc., percent query coverage. Italics indicate >75% query coverage.

c Component of rMenB-OMV vaccine (Novartis).

d Component of the Bexsero and Trumemba vaccines (GlaxoSmithKline/Novartis and Pfizer).

e ND, significant similarity not detected.

**TABLE 3:** Orthologs of N. meningitidis capsule synthesis, transport, and translocation proteins and presence of selected capsule transcripts in N. musculi

Protein query	Query accession no.	Query species	Maximum identity (%)^*a*^	Query coverage (%)^*b*^	Genome annotation	mRNA^*c*^
CssA	WP_002233375.1	N. meningitidis	**72**	*96*	UDP-*N*-acetylglucosamine-2-epimerase	+
CssB	WP_002233374.1	N. meningitidis	**87**	*99*	UDP-*N*-acetyl-d-mannosamine dehydrogenase	ND
CssC	CCP19843.1	N. meningitidis	28	13	Capsule polymerase	ND
CtrA	NP_273135	N. meningitidis	**56**	*93*	Capsule transport complex	+
CtrB	NP_273136	N. meningitidis	**67**	*91*	Capsule transport complex	ND
CtrC	NP_273137	N. meningitidis	**73**	*100*	Capsule transport complex	ND
CtrD	NP_273138	N. meningitidis	**84**	*98*	Capsule transport complex	ND
CtrE	NP_273145	N. meningitidis	**60**	*93*	Capsule translocation	+
CtrF	NP_273146	N. meningitidis	**61**	*99*	Capsule translocation	+

a Boldface, sequence identity > 50%.

b Italics, query coverage > 75%.

c +, positive; ND, not determined.

We also conducted BLAST searches using three β-barrel-containing outer membrane proteins as queries: N. meningitidis NspA, a factor H ligand, and N. gonorrhoeae adhesins OpaD and OpcA. (The N. meningitidis OpcA ortholog is a lectin capable of interacting with vitronectin [40, 41]). NspA and OpaD retrieved the same N. musculi homolog (NspA, 42% identity, 86% query coverage; OpaD, 32% identity, 69% query coverage). The bulk of shared identity in these proteins localized to the β-barrel strands. OpcA did not have a significant hit.

Capsular polysaccharide is a target of several meningococcal vaccines (42). BLAST searches using N. meningitidis capsule proteins showed that N. musculi has genes for capsule biosynthesis, transport, and translocation proteins (Table 3). With the exception of the putative capsule polymerase (CssC), all the capsule-related proteins have high sequence homology with their N. meningitidis orthologs (≥56% identity and ≥75% query coverage).

### N. musculi expresses a polysaccharide capsule.

**FIG 5:**
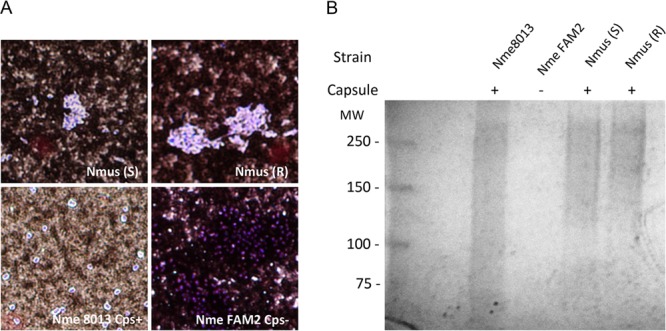
N. musculi (Nmus) produces a capsule. (A) India ink staining of N. musculi smooth (S) and rough (R) strains, capsulated (Cps+) N. meningitidis (Nme) strain 8013, and unencapsulated (Cps−) strain FAM2. The cells were counterstained with crystal violet. (B) Alcian blue staining of lysates of these strains separated by SDS-6% PAGE.

We determined whether N. musculi produces a capsule by using two biochemical tests, India ink and alcian blue staining, that are widely used to detect capsulated organisms (43). After India ink treatment, N. musculi cells (smooth and rough variants) were surrounded by a clear halo against a dark background, which is indicative of capsulated organisms (Fig. 5A). India Ink stained cells of capsulated N. meningitidis 8013 similarly, but not those of the unencapsulated N. meningitidis FAM2 (Fig. 5A). To further confirm that the refractile zone of these cells corresponds to capsular polysaccharide, we stained extracts from the cells with alcian blue (44). The results indicated that a high-molecular-weight alcian blue-reactive smear was present in the capsulated N. meningitidis 8013 and N. musculi AP2365 smooth and rough variants but not in the unencapsulated N. meningitidis FAM2 (Fig. 5B).

Finally, we determined whether capsule genes are transcribed in N. musculi by means of RT-PCR of selected capsule biosynthesis, transport, and translocation genes (see Fig. S5 and Table S2 in the supplemental material). Transcripts for *ccsA*, *ctrA*, *ctrE*, and *ctrF* were detected using this method (Table 3). Taken together, these results indicate that the capsule genes in N. musculi are expressed.

## DISCUSSION

We have developed a genetically tractable small animal model for identifying host and microbial determinants of colonization and persistence. The system pairs the laboratory mouse with a commensal of wild mice, N. musculi, which is closely related to human-dwelling species of Neisseria (17) (Tables 2 and 3) (see below). The protocol does not require antibiotics, hormones, invasive procedures, or genetic manipulation of the animal. A single oral dose of N. musculi results in long-lasting colonization of the oral cavity and gut of the mouse (see Fig. S2 in the supplemental material). All animals were healthy throughout the study.

Using this model, we showed that host genetics and innate immunity strongly control susceptibility to colonization by N. musculi (Table 1). Reagents and tools are readily available for the laboratory mouse, allowing easy dissection of host components that restrict/permit N. musculi colonization. The mice in this study were founder strains of the Collaborative Cross, a powerful new tool that can be used to link genetic traits with biological phenotypes. As susceptibility of these strains to N. musculi colonization ranges from sensitive to highly resistant, the Collaborative Cross will allow us to identify host alleles that determine colonization resistance.

We also used the model to examine the role of the N. musculi type IV pilus in colonization. All Neisseria strains and many bacteria belonging to other genera express Tfp. In the cases of the pathogens N. meningitidis and N. gonorrhoeae, cell culture experiments and a small number of human challenge studies strongly imply a role for the Tfp in colonization (27–31). Here, we corroborate these findings, providing *in vivo* proof that the N. musculi Tfp is an important colonization determinant (Fig. 4). *In vitro* studies have identified other, more subtle activities of the N. gonorrhoeae Tfp, including the reprogramming of the host transcriptional profile and activation of immune signaling pathways (45, 46). Our model provides the first opportunity to identify the *in vivo* endpoints of these activities, as well as the functions of host interaction factors held in common between N. musculi and human commensal Neisseria (Tables 2 and 3).

The animal models currently in use to study N. gonorrhoeae and N. meningitidis are heterologous systems that pair a mouse with a human-specific pathogen (21–23). These approaches limit the ability to utilize the full breadth of mouse genetic techniques available for studying host determinants of persistent colonization. Although N. musculi does not cause disease, it does encode many pathogenic Neisseria host interaction factors and candidate vaccine antigens (Tables 2 and 3). Indeed, N. musculi expresses one of these candidate vaccine antigens, capsular polysaccharide (Fig. 5). Our model will be a useful tool for characterizing the *in vivo* functions of these host interaction factors and is potentially useful for evaluating vaccine candidates for the pathogens.

The fact that N. musculi colonizes the gastrointestinal tract of laboratory mice should not be a surprise. N. musculi colonizes the oral cavity of wild mice and is detected in their guts (17), and Neisseria species have been detected in animal feces (15). Human niches for Neisseria are generally assumed to be the nasopharynx (N. meningitidis, N. gonorrhoeae, and commensal species), genital tract (N. gonorrhoeae and occasionally N. meningitidis), and rectum (N. gonorrhoeae), but to our knowledge studies have not been done to determine the presence of Neisseria in the human gut, either by direct culture or molecular species identification. The large numbers of N. musculi organisms recovered from the oral cavities and guts of mice over a long period indicate the organism is able to adapt to a variety of environments within the animal. Taken together, these observations suggest that Neisseria is a more successful and adaptable organism than had previously been suspected.

There is currently a great interest in the microbiome. In spite of the large number of papers on the subject, little is known about how changes in the microbiome are brought about. Conspicuous by their absence are data concerning the acquisition of a new commensal in the presence of an existing microbiota or after antibiotic treatment. Our model opens the door to these investigations.

## MATERIALS AND METHODS

### Generation of the rifampin-resistant N. musculi strain.

AP2365, a naturally occurring Rif^r^ rough variant of the N. musculi type strain (17), was isolated by plating AP2031 (AP2031^T^) on GCB (Becton Dickinson) agar containing rifampin (50 mg/liter).

### Mouse strains.

All inbred mouse strains and Collaborative Cross parental strains were obtained from the Jackson Laboratory (Bar Harbor, ME). All animal protocols were approved by the University of Arizona IACUC.

### Mouse inoculation protocol.

Mice were rested in the University of Arizona mouse facility for 2 weeks before inoculation. The inoculation protocol is shown in Fig. S1 in the supplemental material. To determine the presence of Neisseria species in the indigenous flora of the animals, the oral cavities of the mice were swabbed using the BD BBL CultureSwab Plus Transport System (Fisher Scientific); the swabs were suspended in GCB medium base (Becton Dickinson) plus Kellogg's supplements I and II (27), and dilutions of the suspensions were plated on GCB agar containing vancomycin (2 mg/liter) and trimethoprim (3 mg/liter). Bacteria on the plates were counted after incubation for 48 h at 37°C, 5% CO_2_. Neisseria has never been recovered from mice before inoculation. Fecal pellets of the mice were suspended and processed similarly. On the day of inoculation, AP2365 was swabbed from an agar plate and resuspended in phosphate-buffered saline (PBS) at an OD_600_ of 2.0. Inbred mice were manually restrained, and 50 μl of the bacterial suspension was pipetted into the oral cavity. The oral cavities of the inoculated mice were swabbed weekly or biweekly. Swab suspensions in GCB medium base (Becton Dickinson) were plated on GCB agar containing rifampin (40 mg/liter), and the plates were incubated for 48 h at 37°C and 5% CO_2_.

### Verification of N. musculi in oral-swab suspensions.

Samples from each colony growing on GCB-rifampin agar were used for verification of N. musculi as described previously. Briefly, internal transcribed spacer (ITS) primers specific to sequences that are highly conserved among Neisseria species were used for colony PCR (17). The ITS sequences of sample isolates were compared to that of the type strain, AP2031, for species validation and were found to be identical.

### Construction of N. musculi Δ*pilE* and its complemented strain.

Table S2 in the supplemental material lists the primers used for strain construction. In AP2365 Δ*pilE*, the *pilE* open reading frame was replaced with a kanamycin resistance cassette. Primers IM011F and IM012R, containing flanking sequences for the *pilE* gene in N. musculi AP2031^T^. were used to amplify the Kan resistance cassette from plasmid pNBNeiKan (17) (synthesized by GenScript). The amplified DNA was purified and transformed into WT N. musculi AP2031^T^ by spot or liquid transformation as described previously, and transformants were selected on GCB agar containing Kellogg's supplements I and II (27) and Kan (50 mg/liter). The Δ*pilE*::*kan* locus in AP2031^T^ was transferred to the rifampin-resistant N. musculi strain AP2365 as follows. Primers NP246F and NP246R2 were used to amplify Δ*pilE*::*kan* from AP2031^T^, and the amplified DNA was cloned into pGEMT (Promega). The recombinant plasmid DNA was introduced into AP2365 by spot transformation. Transformants were selected on GCB agar containing supplements I and II and Kan (50 mg/liter). The Δ*pilE*::*kan* locus in AP2365 was confirmed by Sanger sequencing of PCR products generated with primers NP246F and NP246R2.

The complemented strains AP2365Δ*pilE*::*pilE*_WT_-C10 and AP2365Δ*pilE*::*pilE*_WT_-C21, independent clones, were constructed as follows. Primers IM013 and IM014 were used to amplify the chloramphenicol (Cm) resistance cassette from plasmid pLES94 (47). Primers MR485 and MR486 were used to amplify the WT *pilE* locus in AP2031^T^. The Cm PCR product was digested with Pacl and EcoRV (New England BioLabs), and the *pilE* PCR product was digested with Afel and l KpnI (New England BioLabs). The two digested DNAs were ligated into similarly digested pUC19 (New England BioLabs) using T4 ligase (New England BioLabs). Primers IM0015 and IM0016 were used to amplify the *pilE*::*cm* region in the recombinant plasmid, and the amplified DNA was cloned into pGEMT (Promega). DNA from the resulting plasmid was electroporated into AP2365 Δ*pilE*::*kan* to replace the mutated *pilE* locus. Transformants were selected and maintained on GCB agar containing supplements I and II and chloramphenicol (2.5 mg/liter). The *pilE* loci in the complemented strains were confirmed by Sanger sequencing of the PCR products generated with primers NP246F and NP246R2.

### Transformation assays.

DNA transformations were performed as described previously (17). Briefly, the recipient strains AP2365, AP2365Δ*pilE*, and AP2365Δ*pilE*::*pilE*_WT_-C10 were grown for 16 h at 37°C on GCB agar containing supplements I and II and the appropriate selective antibiotic(s). Bacterial cells were suspended in GCB broth containing MgSO_4_ (5 mM). Thirty microliters of each suspension, previously diluted to an OD_600_ of 1.5, was added to 0.2 ml of liquid GCB containing MgSO_4_ (5 mM) and 1 μg of chromosomal DNA from N. musculi strain AP2093, a naturally occurring isolate whose *rpsL* gene contains a point mutation conferring resistance to streptomycin (17). Following incubation at 37°C for 20 min, the bacteria were added to 2 ml of liquid GCB containing supplements I and II and incubated at 37°C and 5% CO_2_ for 4 h. Transformants were enumerated by plating cells onto GCB agar containing supplements I and II and streptomycin (100 μg/ml), and the total input bacteria were enumerated by plating an equal volume on supplemented GCB agar without antibiotics.

### RNA extraction, cDNA synthesis, and RT-PCR.

Bacterial cells were grown to mid-log phase in GCB broth containing supplements I and II, and total RNA was extracted using TRIzol (Invitrogen) according to the manufacturer's instructions. Contaminating DNA was removed using DNA-free (Ambion). The quality and amount of RNA were determined by spectrophotometry (NanoDrop; Thermo Scientific). For RT-PCR, 1,000 μg of RNA was used to generate the first strand, using Moloney murine leukemia virus (MMLV) reverse transcriptase (Promega) according to the manufacturer's instructions. This was followed by a PCR using GoTaq green master mix (Promega). N. musculi *pilE* was amplified using primers MR489 and MR490. N. musculi 16S rRNA was amplified using primers MR493 and MR494. N. musculi *ctrA* was amplified using primers IM017 and MR018. N. musculi *cssA* was amplified using primers IM019 and IM020. N. musculi *ctrE* was amplified using primers IM021 and IM022. N. musculi *ctrF* was amplified using primers IM023 and IM024. The primer sequences are listed in Table S2 in the supplemental material.

### Growth curves.

Bacterial cells were grown for 16 h at 37°C and 5% CO_2_ on GCB agar containing supplements I and II and the appropriate selective antibiotics. Cells were scraped from the plates, suspended in supplemented GCB, and diluted to an OD_600_ of 0.05; 2 ml of each bacterial sample was added to 60-mm dishes and incubated at 37°C and 5% CO_2_. Bacterial density was measured every 2 h for 10 h using a Beckman Coulter (Brea, CA) DU730 spectrophotometer. The cell density at each time point was expressed by subtracting the OD_600_ value at time zero from the OD_600_ value at the time of collection.

### Adherence assay.

A static biofilm assay adapted from that of Merritt et al. (48) was used to measure adherence. Briefly, 2 ml supplemented liquid GCB was added to each well of a 6-well dish (Corning), and 1 × 10^7^ CFU of N. musculi WT, Δ*pilE*, or the complemented strain was introduced into the wells. The plates were incubated at 37°C and 5% CO_2_ for 16 h. Each well was gently washed 3 times with 1 ml sterile PBS. Any residual wash buffer was forcibly shaken from the plate to remove all planktonic bacteria. One milliliter of 0.1% crystal violet was added to each well, and the plate was incubated for 30 min at room temperature. The excess dye was removed, and all the wells were washed with 10 ml of PBS. Retained crystal violet was solubilized by the addition of 1 ml of 30% glacial acetic acid, and the OD_550_ was measured on a Beckman Coulter (Brea, CA) DU730 spectrophotometer. Three fields were imaged before the initial washes and after crystal violet staining. The results were representative of three independent experiments performed in technical triplicate.

### BLAST searches.

Tblastn searches were conducted using TBLASTN 2.7.1+ (49, 50). Protein query sequences from N. meningitidis and N. gonorrhoeae were used to search N. musculi strain AP2031's genome sequence (PubMLST identifier [ID], 29520 [51]). Many N. meningitidis queries used for the analysis were retrieved from the Protegen protective antigen database (52). The accession numbers for the commensal human-dwelling Neisseria genome data used for BLAST searches were as follows: Neisseria polysaccharea ATCC 43768, NZ_ADBE00000000; Neisseria lactamica 02-06, NC_014752; Neisseria cinerea ATCC 14685, NZ_ACDY00000000; Neisseria subflava NJ9703, NZ_ACEO00000000; Neisseria oralis CCUG 26878, PubMLST ID 19091; Neisseria mucosa ATCC 25996, NZ_ACDX00000000; Neisseria elongata ATCC 29315, NZ_CP007726; Neisseria bacilliformis ATCC BAA-1200, NZ_AFAY00000000.

### India ink stain and light microscopy.

N. meningitidis capsulated strain 8013 and unencapsulated strain FAM2 and N. musculi AP2365^T^ were suspended in India ink (BD Diagnostic) and spread as thin films on a microscope slide (43). After the films were allowed to air dry, the bacteria were counterstained with crystal violet (Gibson) for 1 min. The slides were gently rinsed with water and examined under a light microscope at ×100 magnification.

### Capsule extraction.

Capsule was extracted as described previously (44). N. meningitidis capsulated strain 8013 and unencapsulated strain FAM2 and N. musculi AP2365 rough and smooth variants were grown on GCB agar for 17 to 18 h at 37°C and 5% CO_2_. Cells were suspended in PBS to an OD_600_ of 0.8, and 1 ml of the suspension was pelleted by centrifugation (10,000 × *g*; 5°C) for 2 min. The pelleted cells were resuspended in 0.5 ml PBS and incubated at 55°C for 30 min to allow release of capsular material. The bacteria were pelleted again, and the supernatants were concentrated 10-fold in an Amicon Ultra centrifuge filter with a 10,000-molecular-weight cutoff. Capsular material was separated by SDS-6% PAGE, stained with the cationic dye alcian blue (0.125% alcian blue in 40% ethanol-5% acetic acid; Sigma) for 2 h, and destained overnight in 40% ethanol-5% acetic acid.

## Supplementary Material

Supplemental material
