# Supplementary material for: A Natural Mouse Model for Neisseria Colonization
Source: Infect Immun. 2018 Apr 23;86(5):e00839-17. doi: 10.1128/IAI.00839-17 (PMC5913851; doi:10.1128/IAI.00839-17)
Supplement: Supplemental material [file supp_86_5_e00839-17__index.html]

Supplemental material 

# A Natural Mouse Model for Neisseria Colonization

## Supplemental material

- Supplemental file 1 -

  Fig. S1. Protocol for inoculation and sampling of *N. musculi* in mice.

  PDF, 134K
- Supplemental file 2 -

  Fig. S2. *N. musculi* persistently colonizes the oral cavity and gut of CAST mice.

  PDF, 238K
- Supplemental file 3 -

  Fig. S3. *N. musculi* Δ*pilE* does not produce *pilE* mRNA.

  PDF, 304K
- Supplemental file 4 -

  Fig. S4. OD600 of cultures of *N. musculi* WT, Δ*pilE*, and complemented strain Δ*pilE*::*pilE*WT-C10.

  PDF, 192K
- Supplemental file 5 -

  Fig. S5. Transcripts of *ctrA*, *cssA ctrE*, and *ctrF* are detected in *N. musculi*.

  PDF, 294K
- Supplemental file 6 -

  Table S1. Transformation frequencies of *N. musculi* WT, Δ*pilE*, and complemented strain AP2365Δ*pilE*::*pilE*WT-C10.

  PDF, 382K
- Supplemental file 7 -

  Table S2. Primers used in this study.

  PDF, 95K
- Supplemental file 8 -

  Table S3. *P* values for the frequency of colonization of CAST and C57BL/6J by *N. musculi* WT, Δ*pilE*, and complemented strain AP2365Δ*pilE*::*pilE*WT-C10 using the Mantel-Cox rank order test.

  PDF, 285K
