# Supplementary material for: A Natural Mouse Model for Neisseria Colonization
Source: Infect Immun. 2018 Apr 23;86(5):e00839-17. doi: 10.1128/IAI.00839-17 (PMC5913851; doi:10.1128/IAI.00839-17)
Supplement: Supplemental material [file IAI.00839-17_zii999092381s1.pdf]

SUPPLEMENTAL FIGURE 1

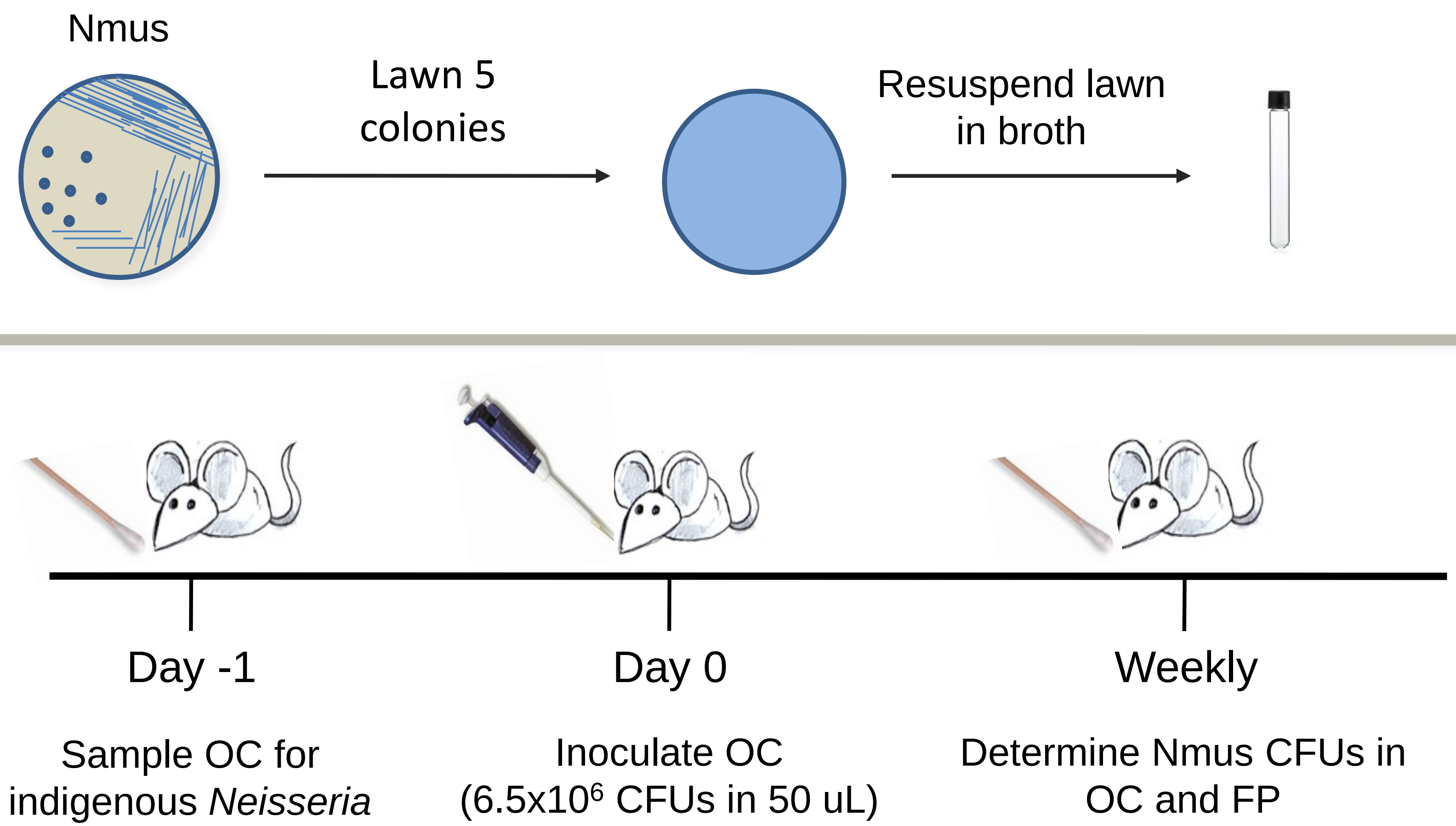

SUPPLEMENTAL FIGURE 1. Protocol for inoculation and sampling of *N. musculi* in mice. CFU: colony forming units; OC: oral cavity; FP: fecal pellet.
