# Supplementary material for: A Natural Mouse Model for Neisseria Colonization
Source: Infect Immun. 2018 Apr 23;86(5):e00839-17. doi: 10.1128/IAI.00839-17 (PMC5913851; doi:10.1128/IAI.00839-17)
Supplement: Supplemental material [file IAI.00839-17_zii999092381s2.pdf]

SUPPLEMENTAL FIGURE 2

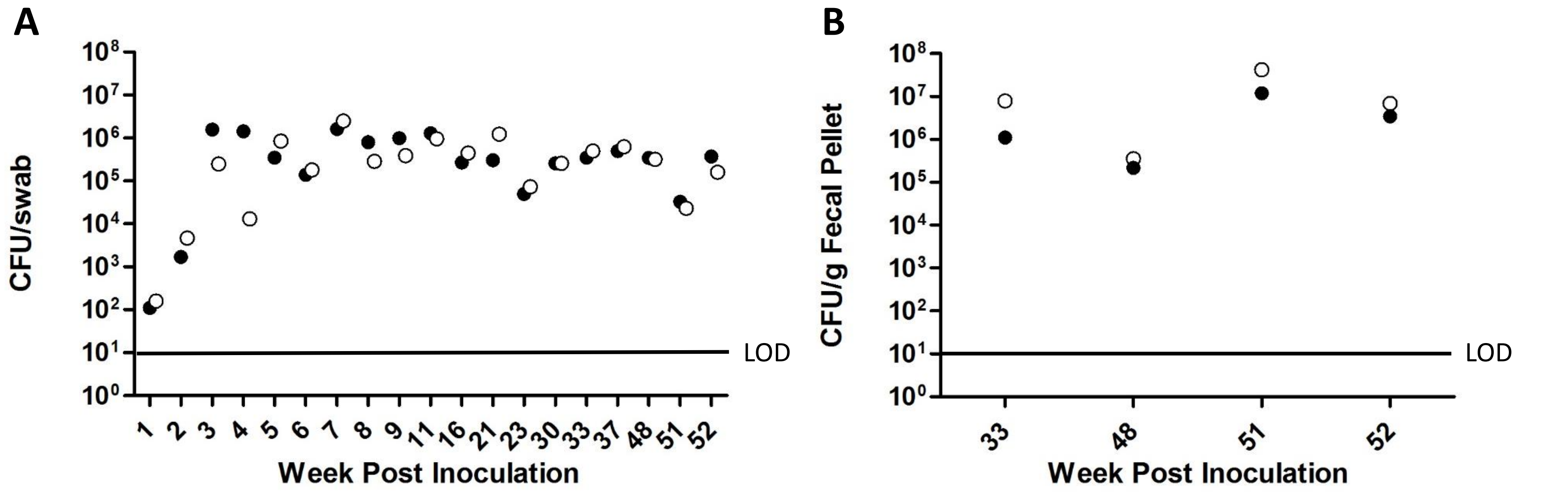

SUPPLEMENTAL FIGURE 2. *N. musculi* persistently colonizes the oral cavity (A) and gut (B) of CAST mice. LOD: limit of detection.
