# Supplementary material for: A Natural Mouse Model for Neisseria Colonization
Source: Infect Immun. 2018 Apr 23;86(5):e00839-17. doi: 10.1128/IAI.00839-17 (PMC5913851; doi:10.1128/IAI.00839-17)
Supplement: Supplemental material [file IAI.00839-17_zii999092381s3.pdf]

SUPPLEMENTAL FIGURE 3

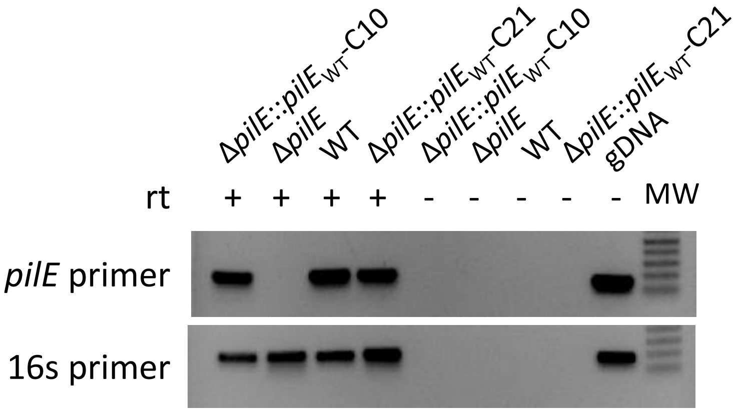

SUPPLEMENTAL FIGURE 3. Nmus Δ*pilE* does not produce *pilE* mRNA. WT: parental wild type *N. musculi*; AP2365Δ*pilE*::*pilE*$_{WT}$-C10 and AP2365Δ*pilE*::*pilE*$_{WT}$-C21: *pilE* complemented strains; rt: reverse transcriptase; gDNA: genomic DNA control.
