# Supplementary material for: A Natural Mouse Model for Neisseria Colonization
Source: Infect Immun. 2018 Apr 23;86(5):e00839-17. doi: 10.1128/IAI.00839-17 (PMC5913851; doi:10.1128/IAI.00839-17)
Supplement: Supplemental material [file IAI.00839-17_zii999092381s4.pdf]

## SUPPLEMENTAL FIGURE 4

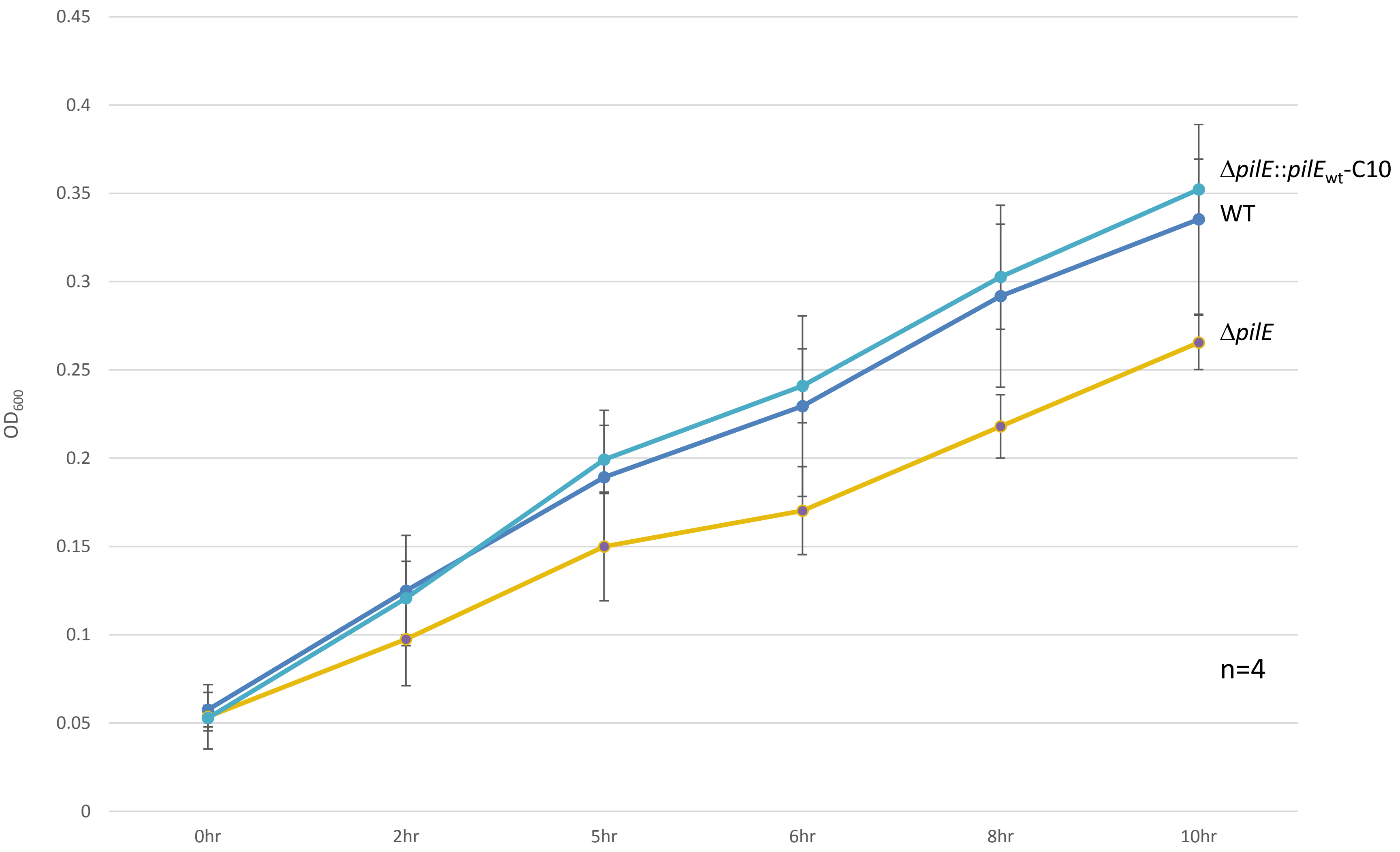

**SUPPLEMENTAL FIGURE 4**. $OD_{600}$ of cultures of *N. musculi* WT, ∆*pilE* and complemented strain ∆*pilE*::*pilE*$_{wt}$-C10. Values are the average of 4 independent experiments.
