# Supplementary material for: A Natural Mouse Model for Neisseria Colonization
Source: Infect Immun. 2018 Apr 23;86(5):e00839-17. doi: 10.1128/IAI.00839-17 (PMC5913851; doi:10.1128/IAI.00839-17)
Supplement: Supplemental material [file IAI.00839-17_zii999092381s5.pdf]

SUPPLEMENTAL FIGURE 5

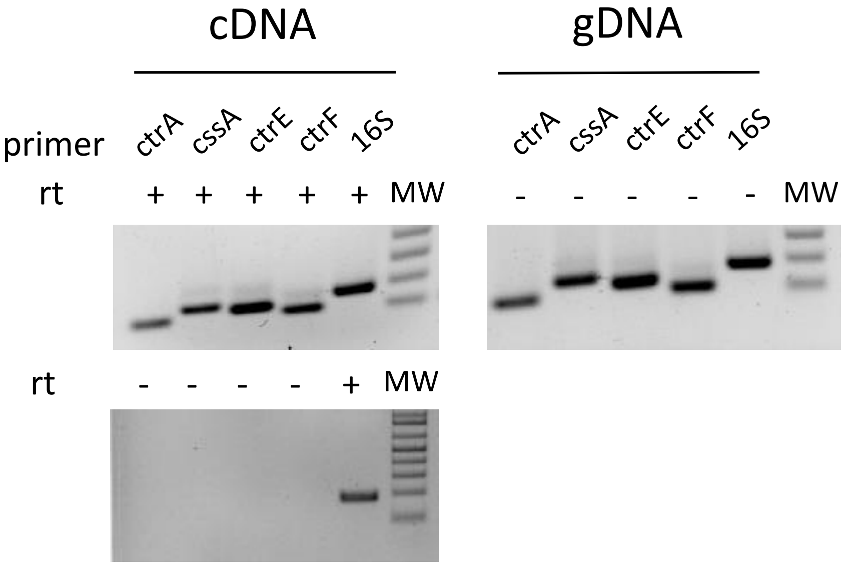

SUPPLEMENTAL FIGURE 5. Transcripts of *ctrA, cssA ctrE* and *ctrF* are detected in *N. musculi*. rt: reverse transcriptase; cDNA: complementary DNA; gDNA: genomic DNA control.
