# Supplementary material for: A Natural Mouse Model for Neisseria Colonization
Source: Infect Immun. 2018 Apr 23;86(5):e00839-17. doi: 10.1128/IAI.00839-17 (PMC5913851; doi:10.1128/IAI.00839-17)
Supplement: Supplemental material [file IAI.00839-17_zii999092381s6.pdf]

SUPPLEMENTAL TABLE 1. Transformation frequency of Nmus WT, Δ*pilE* and complemented strain AP2365Δ*pilE*::*pilE*$_{WT}$-C10.

**Strain**	**DNA**	**Transformation frequency [a]**
AP2365 (WT)	AP2098-Sm^R gDNA [c]	$6.16 \times 10^{-4} \pm 3.19 \times 10^{-4}$
AP2365Δ*pilE*	AP2098-Sm^R gDNA	$<6.98 \times 10^{-7} \pm 3.06 \times 10^{-8}$ [b]
AP2365Δ*pilE*::*pilE*$_{WT}$-C10	AP2098-Sm^R gDNA	$8.94 \times 10^{-5} \pm 1.93 \times 10^{-5}$
AP2365 (WT)	No DNA	$<6.42 \times 10^{-8} \pm 1.79 \times 10^{-8}$ [b]
AP2365Δ*pilE*	No DNA	$<7.03 \times 10^{-7} \pm 6.71 \times 10^{-8}$ [b]
AP2365Δ*pilE*::*pilE*$_{WT}$-C10	No DNA	$<2.21 \times 10^{-7} \pm 2.79 \times 10^{-9}$ [b]

[a]Transformation frequency is expressed as the number of Sm^R CFU/total CFU. Values are the mean of 3-5 independent experiments ± SEM. [b]Limit of detection. [c]Genomic DNA from AP2098, a naturally-occurring Streptomycin resistant *N. musculi* isolate.
