# Supplementary material for: A Natural Mouse Model for Neisseria Colonization
Source: Infect Immun. 2018 Apr 23;86(5):e00839-17. doi: 10.1128/IAI.00839-17 (PMC5913851; doi:10.1128/IAI.00839-17)
Supplement: Supplemental material [file IAI.00839-17_zii999092381s7.pdf]

SUPPLEMENTAL TABLE 2. Primers used in this study.

Primer pairs	Use	Sequence
IM011F*	To delete *pilE*	**GAGGCCAAGCCCAAGCCCAAGCCC AGGCAGAAGCAGCAGCCAAAAGCG GCAAGCTGTAAATTCCTTCTTCTTTT GATTGCAGTACAACGTGCCAAGCAC ATTACGGTTTTACAACTATATAAATTT TATTCACTTTTAAACTGGAGTTTTAA C**CTCGAGGGCTTGACACTTTATG
IM0012R*		**CAGCGTTGTTTTATTTTGTGTATTTAG GTGATACCTTCCAATAAGGCATCAGT CCAAACCCTGTGCGGTAGTTGCCTG TATCGGATAAAAGCGCACCATATTG CTATAGTGCGCTTTTCGGTTAAACCT TAAAAGTTATTAAGGTTGTTGA**ATCG ATGTTTAAACTTCAGACGGC
NP246F	Detect *pilE* mutant	TGAAACACAAGGCCGTCTGA
NP246R2		TGACTTAAACGACTTTTTCTCATAGGG
IM013	Amplify *CmR* gene	atcatc**ttaattaa**CTGCCACTCATCGCAGT A
IM014		atcatc**gatatc**GGGATGCATAAACTGCAT CCCGAGATTTTCAGGAGCTAAGGAAG
MR485	To complement *pilE*	atcatc**agcgct**GCAAGGCCGTCTGAAACAC
MR486		atcatc**ggtacc**CTGTGCGGTAGTTGCCT GTA
IM015	Amplify *pilE*-CmR	GCTGCAAGGCCGTCTGAAACAC
IM016		TACAGCCCCTAAAGTTAAGCCTGCCG TGTTTCAAATGCGG CATACGCTTTATCGGCGTTTCTGCCA GAATTTCCCGAATGTTTCACTCTTGC CGCTGCATCGGCAGCGTTGTTTTATT TTGTGTATTTAGGTGATACCTTCCAAT AAGGCATCAGTCCAAACCCTGTGCG GTAGTTGCCTGTCTGCCACTCATCGC AGTA
MR493		ATCCTGGCTCAGATTGAACG

MR494	Detect *16S* transcript	CCGCTTTCCTTCTCAAAGTG
MR489<br>MR490	Detect *pilE* transcript	GGCTTTACCCTGATCGAGTTG<br>CCGTCTAAAACGCAGGTTTC
IM017<br>IM018	Detect *ctr*A transcript	AGTATCCGTATGCCGCTCAC<br>AGCTGCACCGAAATATCCTG
IM019<br>IM020	Detect *cssA* transcript	CAGATGGACGTTGTGTTTGC<br>AGGGAAAATTTCGGAGAAGG
IM021<br>IM022	Detect *ctrE* transcript	CATAAAAAGGCAGGCCGTAG<br>GACCAAACCGTAACCGAATG
IM023<br>IM024	Detect *ctrF* transcript	AGCTGCCTTGAAAGGTGATG<br>GTTACGCTCAACAGCACCAG

*The regions of primers IM011F and IM0012R that anneal to the *pilE* locus of AP2031 are in bold.
