# Supplementary material for: A Natural Mouse Model for Neisseria Colonization
Source: Infect Immun. 2018 Apr 23;86(5):e00839-17. doi: 10.1128/IAI.00839-17 (PMC5913851; doi:10.1128/IAI.00839-17)
Supplement: Supplemental material [file IAI.00839-17_zii999092381s8.pdf]

SUPPLEMENTAL TABLE 3. *p*-values for the frequency of colonization of CAST and C57BL/6J by *N. musculi* WT, Δ*pilE* ,and complemented strain AP2365Δ*pilE*::*pilE*$_{WT}$-C10 using Mantel-Cox Rank Order Test.

	**WT vs Δ*pilE***	**WT vs Δ*pilE*::*pilE*$_{WT}$-C10**	**Δ*pilE* vs Δ*pilE*::*pilE*$_{WT}$-C10**
CAST-OC [a]	<0.0001	0.3316	<0.0001
CAST-FP [b]	<0.0001	0.9116	<0.0001
C57BL/6J-OC [a]	<0.0001	0.0013	0.0030
C57BL/6J-FP [b]	<0.0001	0.0008	0.3316

(a) OC, Oral cavity. (b) FP, Fecal pellet.
